# The clinical characteristics and outcomes of patients with pulmonary hypertension in association with hyperthyroid state: A systematic review

**DOI:** 10.1097/MD.0000000000029832

**Published:** 2022-06-30

**Authors:** Fateen Ata, Adeel Ahmad Khan, Zohaib Yousaf, Hassan Choudry, Areej Marwan Mohammed, Bilal Ahmed, Ahmed Muaaz Umer, Fareeha Khan, Dabia Hamad SH Al Mohanadi, Emad Naem, Muhammad Zahid

**Affiliations:** a Department of Internal Medicine, Hamad General Hospital, Hamad Medical Corporation, Doha, Qatar; b Department of Endocrinology, Hamad General Hospital, Hamad Medical Corporation, Doha, Qatar; c Department of Respiratory Medicine, University Hospital of Leicester, England; d University Hospitals Birmingham NHS Foundation Trust, Birmingham, England; e Research Fellow, Institute of Molecular Cardiology Department of Cardiovascular Medicine, University of Louisville Kentucky, USA; f Department of Public Health, The University of Manchester, England; g Weill Cornel Medicine, Qatar.

**Keywords:** grave disease, hyperthyroidism, pulmonary hypertension, thyroid, thyroiditis

## Abstract

Pulmonary hypertension (PHTN) may occur in thyroid disorders, especially in hypothyroidism. However, there is increasing evidence of PHTN in hyperthyroidism (HTH). The etiology, clinical course, management, and factors associated with outcomes of PHTN in the setting of HTH are unascertained. This systematic review consolidates available evidence on patients with HTH who developed PHTN.

**Methods:**

We conducted a systematic review on English articles from PubMed, Scopus, and Google Scholar reporting PHTN in patients with hyperthyroidism. Data were analyzed and reported in Microsoft Excel 2020, SPSS version 26, and Jamovi version 1.2.

**Results:**

We identified 589 patients with PHTN in the setting of HTH. Etiologies included Grave disease 66.7%), toxic multinodular goiter (TMNG) (16.8%), drug-induced HTH (0.3%), thyroiditis(0.8%), and toxic adenoma(0.1%). Most patients did not receive any specific management for PHTN and were managed by antithyroid treatment (97.4%). Outcomes of PHTN were reported in 181 patients, with a 94% recovery rate. Pulmonary artery pressures (PAP) before and after HTH management ranged from 22.5 to 75 mm Hg and from 24 to 50 mm Hg, respectively. Outcome analysis performed on data from case reports and series with individually identifiable data revealed a 67.6% female preponderance. An estimated 73.5% of the patients had PHTN at the initial presentation of HTH, which was associated with a better resolution rate of PHTN(OR: 12, *P*-value: 0.048). TRAB was positive in 47% patients with no clinical difference in outcomes. antiTG AB was reported positive in 29.4%, all of whom had an improvement, compared to an 83.3% improvement rate in those with negative antiTG AB. Various etiologies and treatments did not have any significant differences in the outcome of PHTN.

**Conclusions:**

PHTN can be present at the initial diagnosis of HTH, which is associated with better outcomes of PHTN. There is a clear female preponderance in the development of PHTN. However, resolution rates seem to be better in males. Although TRAB is associated with the development of PHTN, it does not seem to affect the outcomes. PHTN in patients with HTH does not need any specific management, with >90% resolution with antithyroid therapy. Whether any specific antithyroid therapy has a better outcome in PHTN needs to be explored prospectively.

## 1. Introduction

Alterations in the serum thyroid hormone levels are associated with significant pathophysiologic changes in various organ systems, including the cardiovascular system.^[1]^ These include arrhythmias, atrial fibrillation (Afib), thromboembolism, heart failure, cardiomyopathies, valvular heart disease, and pulmonary hypertension (PHTN).^[2]^ Although the association of PHTN with thyroid disorders is known, the mechanism and dynamics of the association remain largely unexplored. PHTN is a pathologic elevation in the mean pulmonary arterial pressure (>20 mm Hg) and a high pulmonary vascular resistance (*>*3 WU), resulting in right-sided heart failure.^[2,3]^ It is a relatively uncommon cardiovascular pathology and is usually seen in adult females, with an incidence of 15–50 per million population.^[4]^ However, the 5-year mortality rate with PHTN is reported to be as high as 61.5%.^[5]^ It usually presents as exertional dyspnea, fatigue, chest pain, and lower limb edema.^[4]^ Diagnosis is made on echocardiographic findings and cardiac catheterization, which is the gold standard investigation even though invasive.^[4]^ PHTN is divided into 5 groups based on etiologies and different pathophysiologic mechanisms of development of PHTN.^[3]^ Thyroid disorders are included in the etiology of group 5 PHTN (PHTN with unclear cause).^[3]^ Treatment of PHTN depends on its etiology and severity. Most of the specific pharmacological treatment evidence is available for Group 1 PHTN (pulmonary arterial hypertension), including calcium channel blockers, endothelin receptor antagonists, phosphodiesterase type 5 inhibitors, guanylate cyclase stimulators, and prostacyclin drugs. Managing the underlying etiological pathology is the cornerstone in the treatment of other groups of PHTN.^[6]^ Lung transplantation is the ultimate treatment for patients with end-stage and refractory disease.^[7]^

Among thyroid disorders, PHTN is more common in autoimmune hypothyroidism compared to HTH. However, the occurrence rates vary in studies. Miura et al reported hypothyroidism in 81% and HTH in 19% of their patient population with PHTN.^[8]^ The prevalence of PHTN in HTH is reported to be around 35% to 47%.^[9,10]^ However, this prevalence is yet to be validated in large cohorts. Treatment of HTH usually results in resolution or improvement in PHTN.^[2]^ Most of the literature comprises case reports, series, and small sample cohorts, with variable results. There are multiple retrospective studies, but mostly with small sample sizes; hence, the results have not been consistent.^[9–24]^ Some studies have shown an association of hormone levels such as free thyroxine (FT^[4]^), and antibodies such as TRAB with increased pulmonary pressures, whereas other studies did not find this association.^[12,15,16,18–20]^ Additionally, the pathophysiology of PHTN in HTH remains unclear. Most of the studies argue PHTN to be secondary to HTH. A multifactorial mechanism is proposed involving direct effects of thyroid hormones on pulmonary vascular proliferation, chronotropic effects of the hormones on the cardiovascular system, and autoimmune mediated endothelial dysfunction.^[1]^ However, in 1 retrospective study, the authors concluded thyrotoxicosis to be due to preexisting PHTN and its treatment with epoprostenol.^[13]^ Lastly, most of the data come from cohorts of patients with thyroid disorders, focusing on factors associated with the development of PHTN. There is very limited data on the cohort of hyperthyroid patients with PHTN, focusing on its clinical characteristics and management. To understand these uncertainties in clinical characteristics and management of PHTN in HTH, we aimed to combine the available data in a systematic review to produce cumulative evidence and derive more robust conclusions.

## 2. Materials and methods

### 2.1. Literature search

We conducted a systematic literature search from any date up to 14^th^ May 2021 using various electronic databases (PubMed, Scopus, Google Scholar) to identify English language articles to be added in our review. The following search term was used: “pulmonry hypertension” AND “thyroid” OR “hyperthyroid*” OR “thyroiditis” OR “Graves”.

Our predefined PECO question was:

Population: Pediatric and Adult patients with a diagnosis of hyperthyroidism.

Exposure: Presence of pulmonary hypertension.

Control: None

Outcomes: Resolution, improvement, progression of or persistent pulmonary hypertension, and death.

### 2.2. Study selection and bias assessment

Studies that were identified from the above search strategy were exported to Endnote for further screening. Two reviewers independently screened the articles by title, abstract, and keywords. The extracted articles were screened by title and abstract, following an in-depth screening of the relevant studies. The quality of the case reports and series were analyzed by using the Joanna Briggs Institute case report appraisal checklist for inclusion in systematic reviews.^[25]^ The quality of evidence in larger observational studies was assessed using the Grading of Recommendations, Assessment, Development, and Evaluations (GRADE) approach and Methodological index for nonrandomized studies (MINORS) assessment tool.^[26,27]^ In case of disagreements in quality assessment and article screening, a third reviewer independently analyzed the disputed articles to reach a conclusion.

**Figure 1.**
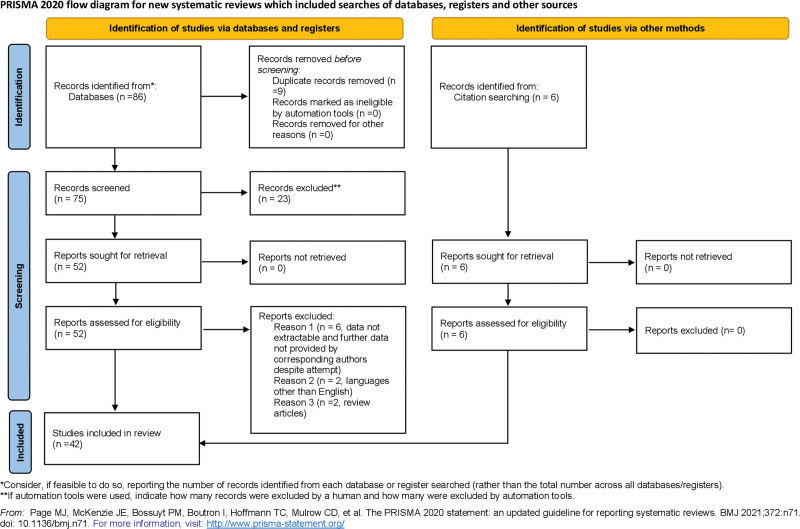
PRISMA flow diagram of the article screening process.

Eligible studies (N = 52) reported the presence of PHTN in patients (of all ages) with a hyperthyroid state secondary to various etiologies of HTH. The studies included case reports, case series, retrospective, and prospective observational studies (Fig. 1). All of the articles stated that secondary causes of PHTN were ruled out. In 1 case the patient had mild COPD, but investigation revealed normal spirometery and hence the authors attributed PHTN not due to mild COPD but HTH.^[28]^ We did not find any relevant clinical trials. Articles that were not original were excluded from the review. Articles that reported patients having PHTN prior to the development of HTH were also excluded from the review. Additionally, articles that reported PHTN in hypothyroid patients were also excluded from the review.

### 2.3. Data collection and statistical analysis

Socio-demographic variables, clinical (laboratory and radiological details) variables concerning HTH and PHTN, treatments, and outcomes were reported in all the cases subject to availability. Laboratory values mentioned as less than a specific number were written as the highest possible number for the purpose of analysis (e.g., <0.1 was written as 0.09). Outcome analysis was performed on PHTN with either complete resolution (evident by symptom resolution and follow-up TTE with normalization of PAP to <25 mmHg), improved PHTN (reduction in PAP in follow-up visit with symptom resolution or improvement), persistent PHTN (evident by symptom persistence and follow-up TTE with no reduction in PAP posttreatment), and death. Data were recorded and analyzed in Microsoft Excel 2020, Jamovi version 1.2 (created in 2020, Sydney, Australia), and SPSS 26. Descriptive and summary statistics were used to report the overall data of 589 patients. 30 studies with 34 patients (26 case reports and 4 case series) reported characteristics and data of individual patients. This data was analyzed using Chi square test for categorical variables and Mann–Whitney *U* test for associations of continuous variables, with regards to outcomes of PHTN.

## 3. Results

A total of fifty-two articles were identified relevant to the research question. However, 6 articles were excluded from the review as most of the data were either not available or not extractable.^[12,21,24,29–31]^ Furthermore, 2 articles were in language other than English and 2 were review articles without original data and were removed from the analysis. Forty-two studies with 589 patients were included in the review, comprising 9 retrospective studies, 3 prospective studies, 4 case series, and twenty-six case reports.

### 3.1. Patient demographics

**Figure 2.**
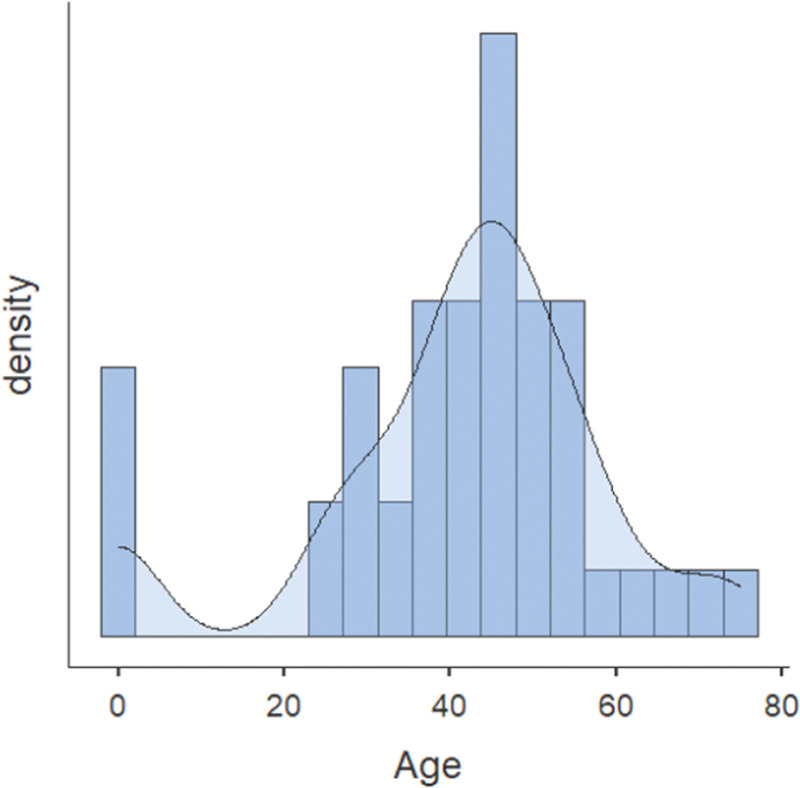
Density histogram describing the distribution of patients’ age (in years).

We found patients from a wide age range who had HTH with PHTN. The youngest patient was 3 days old, whereas the oldest patient was 75. The majority of reported patients were between 40-60 years (Fig. 2). Most of the patients were females (N=374, 63.4%), whereas males comprised 29.3% of the patient population (N=173). Gender was not specified in 42 patients. HTN was the commonest reported comorbidity (N=50, 8.4%), followed by diabetes mellitus (DM) (N=14, 2.3%), heart failure (N= 5, 0.8%), coronary artery disease (CAD) (N= 4, 0.6%), chronic kidney disease (CKD) (N= 1, 0.1%), and chronic obstructive pulmonary disease (N= 1, 0.1%).

### 3.2. Clinical features of HTH

Symptoms of HTH were not reported in a majority of cases. Among the available signs and symptoms, shortness of breath (SOB) was the commonest reported feature (N = 25, 4.2%), followed by Afib (N = 20, 3.3%), palpitations (N = 18, 3%), tremors (N = 14, 2.3%), weight loss (N = 12, 2%), ophthalmopathy (N = 12, 2%), diarrhea (N = 9, 1.5%), sweating (N = 6, 1%), and heat intolerance (N = 3, 0.5%). Goiter was present in 15 patients (2.5 %). TSH values ranged from 0 to 0.09 mU/L. T3 and T4 levels ranged from 0.5-771 ng/dL and 1.28-20,800 ng/dL, respectively. The most commonly reported positive antibody was TRAB (N=283, 48%), followed by Anti TPO (N = 272, 46.1%), and lastly anti-nuclear antibody (ANA) which was positive in 10 patients (1.69%).

Although various etiologies were reported for HTH, Grave disease was most prevalent (N = 393, 66.7%). This was followed by toxic multinodular goiter (TMNG) (N= 99, 16.8%), thyroiditis (N = 5, 0.8%), drug-induced HTH (N = 2, 0.3%), and toxic adenoma (N = 1, 0.1%). No specific etiology was reported in 89 patients (15.1%).

Most of the patients were managed medically, with thionamides (N = 262, 44.4%). Fifty-4 patients (9.1%) underwent thyroidectomy, whereas 34 patients (5.7%) had radio ablation of the hyperactive thyroid gland. Treatment was not specified in 40.5% of patients. Five patients (0.8%) were reported to have failed first-line management and eventually responded to second-line treatment (evident by euthyroid status postsecond-line treatment). The outcome of HTH was reported in 198 patients (33.6%). Among these, 89.8% (N = 178) achieved euthyroid status, whereas 20 patients (10.1%) had persistent HTH.

### 3.3. Clinical features of PHTN

Among the 589 patients, PHTN was present at the initial presentation of HTH in 83 patients (14%). Dyspnea was the most frequently reported symptom of PHTN (N = 108, 18.3%), followed by peripheral edema (N = 15, 2.5%), anorexia (N = 3, 0.5%), cough (N = 3, 0.5%) ¸ abdominal pain (N = 2, 0.3%), chest pain (N = 1, 0.1%), and lastly syncope (N = 1, 0.1%). The diagnosis of PHTN was established via echocardiogram in all of the patients (N = 589). Cardiac catheterization was performed to confirm the diagnosis of PHTN in 3 patients (0.5%).

Most of the patients did not receive any specific treatment for PHTN (N = 574, 97.4%). However, a few patients (N = 15, 2.5%) were reported to have a specific treatment for PHTN. Diuretics were reported in 12 patients (2%). The use of nitrous oxide and calcium channel blockers was reported in 2 and 1 patients, respectively. We did not find any patients treated with endothelin receptor antagonists, phosphodiesterase-5 inhibitors, or prostacyclin receptor agonists.

**Figure 3.**
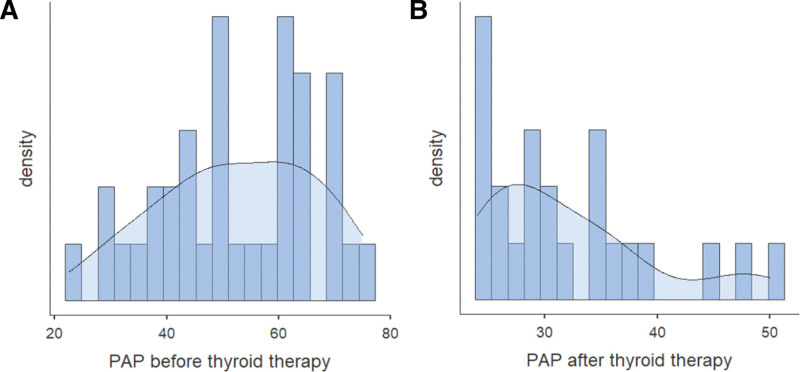
Density histogram describing the distribution of PAP (mm Hg) before and after HTH treatment.

Outcomes of PHTN were reported in 181 patients. We collected pulmonary artery pressures (PAP) and Right Ventricular Systolic Pressures (RVSP) before and after HTH treatment. PAP before HTH management ranged from 22.5 to 75 mm Hg, whereas posttreatment PAP ranged from 24 to 50 mm Hg (Figure 3A, B). RVSP before therapy was 60-80 mm Hg, whereas it dropped down to 35 to 51 mm Hg after HTH management.

Out of 181 patients with reported outcomes, a complete resolution of PHTN was reported in 98 patients (54.1%), whereas improvement in PHTN was reported in 72 patients (39.7%). Overall, a positive outcome was present in 94% of patients with reported outcomes. The persistence of PTHN was rare and was reported in only 9 patients (4.9%).

### 3.4. Mortality

A total of 2 patients died. The first patient was 45 years old female who presented with a thyroid storm and was diagnosed with PHTN during admission. She died due to a right temporoparietal infarct with hemorrhagic transformation.^[32]^ The exact details and the cause of death was not mentioned in the other patient (the patient was a part of a retrospective study with cumulative data).^[22]^

### 3.5. Outcome analysis

We did outcome analysis on the data from case reports and series in order to understand associations of various variables with the resolution of PHTN post HTH treatment. We could not add data from larger studies (including supplementary files). The corresponding authors of these studies were contacted to get the individualized data of the patients, however we were not able to acquire the data. Therefore, these studies were excluded from the outcome analysis as the cumulative data made it difficult to isolate the information for an accurate analysis. An attempt was made to For the purpose of analysis, wherever a variable was not mentioned as present, it was considered absent. Out of 34 patients included in the outcome analysis, 23 (67.6%) were females, whereas 10 (29.4%) were male, and gender was not specified in 1 patient. All male patients had an ultimate improvement in PHTN posttreatment, whereas the improvement rate in females was 82.6%. The difference was not statistically significant. Nineteen patients (55.8%) had a complete resolution of PHTN after HTH treatment, whereas 11 patients (32.3%) had improvement in PHTN after treatment of HTH. We combined resolved and improved into a single outcome of improved (N = 30, 88%) for analysis. Persistent or worsening of PHTN was reported in only 4 patients (11.7%), out of whom 1 died.

PHTN was present at the initial presentation of HTH in 25 patients (73.5%). Among these, 24 patients (96%) had improvement in PHTN posttreatment, compared to a 66.6 % improvement in those who had a diagnosis of HTH preceding PHTN (OR: 12, *P*-value: 0.48). Among the various comorbidities reported, hypertension was the most common 1, reported in 5 patients (14.7%), out of which 4 patients (80%) improved, comparted to 89.6% improvement of PHTN in those who did not have HTN reported as a comorbidity. Among the presenting symptoms of PHTN, chest pain was reported in 1 patient, who did not improve, compared to a 90.9% improvement rate in patients without chest pain. Other symptoms included dyspnea (70.5%), peripheral edema (44.1%), anorexia (8.8%), cough (8.8%), abdominal pain (5.8%), and syncope (2,9%). However, none of them had a statistically significant correlation to the outcomes of PHTN.

TRAB was positive in 16 patients (47%) with PHTN, out of whom 14 (87.5%) improved posttreatment, compared to a 88.8% improvement rate in patients with negative TRAB. Anti TG AB was reported positive in 10 patients (29.4%), all of whom had an improvement, compared to an 83.3% improvement rate in those with negative Anti TG AB. Anti TPO AB was reported positive in 12 patients (35.2%), out of which 11 improved. However, the differences were not statistically significant.

Grave disease was the most common etiology (N = 27, 79.4%) behind HTH in this subgroup. Drug-induced HTH was the etiology in 2 patients. Toxic adenoma was the cause of HTH in 1 patient. The exact cause of HTH was not specified in 4 cases. In the outcome analysis, none of the etiologic causes of HTH had a significant correlation to the improvement of PHTN after treatment.

Radioactive Iodine Uptake (RIU) was present in 14 patients (41.1%) and negative (or not reported) in 20 (58.8%). Thirteen patients (92.8%) with RIU had an improvement in PHTN, compared to 90% improvement rate in patients with negative (or not reported) RIU upon diagnosis of HTH. Out of 33 patients who received thionamides, 24 (72.7%) achieved euthyroid state, whereas 31 (93.9%) had an improvement in PHTN (normalization or decreased PAP posttreatment). Out of the 12 patients who underwent radioiodine ablation, 91.6% achieved euthyroid status, and 100% showed improved PHTN (complete or partial). Lastly, 2 patients underwent thyroidectomy, and both achieved euthyroid state as well as resolution of PHTN after surgery

The data of 3 included prospective studies is separately tabulated (Table 2).

## 4. Discussion

We present the most extensive systematic review on hyperthyroid patients who had PHTN as a complication or manifestation of HTH. There are multiple studies on cohorts of hypothyroid and hyperthyroid patients, among which a proportion are reported to have PHTN. These studies report the prevalence of PHTN in patients with thyroid disorders in general. However, only a few studies have been conducted specifically on hyperthyroid patients with PHTN, aiming to understand the combination’s clinical characteristics, management, and outcomes. Our outcome analysis showed that PHTN was presenting feature in 73.5% of patients. The presence of PHTN at the initial presentation of HTH possibly has better overall outcomes concerning PHTN (OR: 12, *P*-value: 0.048). Hence, it is clinically significant to identify HTH as the cause of patients presenting with PHTN to start the treatment of HTH promptly. This finding can further be confirmed in larger cohorts where the timeline of initiation of PHTN and HTH are noted and correlated to the outcomes. The presence of TRAB does not seem to affect outcomes of PHTN. However, the presence of antiTG AB may positively impact the clinical outcome of PHTN. In our review, 97.4% of patients were not treated explicitly for PHTN, and the management was based on antithyroid therapies.

**Figure 4.**
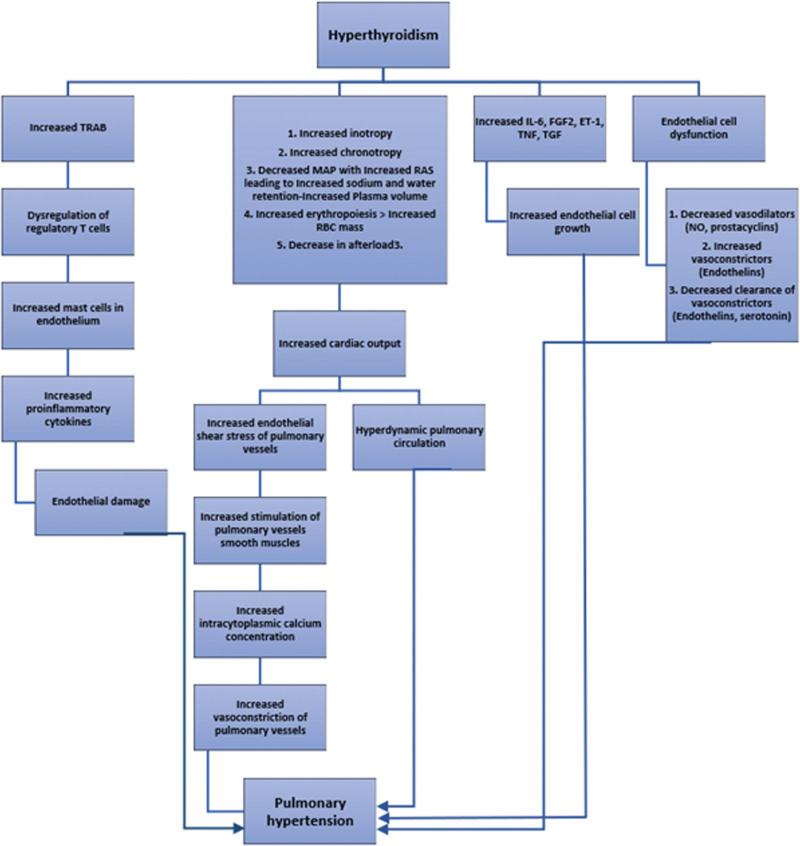
Proposed pathophysiology of development of PHTN in patients with HTH, adapted from Scicchitano et al and Vallabhajosula et al.^[1,33]^ ET-1: Endothelin-, FGF2: Fibroblast growth factor-2, IL-6: Interleukin-6, TNF: Tumor necrosis factor, TGF: Transforming growth factor, MAP: Mean arterial pressure, NO: Nitric oxide, RAS: Renin-angiotensin system, RBC: Red blood cells, TRAB: TSH receptor antibody.

HTH causes increased cardiac output through various mechanisms, such as direct chronotropic effects on the heart, increment in the blood volume, and decreased systematic resistance, leading to a decreased afterload.^[33]^ However, the pathophysiology behind the development of PHTN in patients with HTH remains unknown. Broadly, thyroid disorders can result in PHTN in 2 ways, directly affecting the pulmonary vascular system and contributing to other medical conditions, which can ultimately lead to PHTN.^[33]^ Scicchitano et al have proposed multi-mechanistic pathophysiology behind the development of PHTN in patients with HTH.^[1]^ The direct effects of raised thyroid hormones cause a hyperdynamic pulmonary blood flow via an increased cardiac output. This produces shear stress on the pulmonary vasculature, resulting in an increased production of smooth muscle cells in the walls of the pulmonary vessels. The shear stress also causes loss of endothelial caveolin-1, which decreases calcium influx into the cells. This results in endothelial dysfunction and ultimately smooth muscle cell proliferation through feedback mechanism.^[1]^ Another mechanism by which pulmonary vasculature shear stress causes PHTN is by activating vascular smooth muscle cell mechanosensitive channels. This leads to an increase in the cytosolic calcium level, resulting in pulmonary vascular vasoconstriction. Additionally, excess free T3 and T4 in the systemic circulation triggers an amplification of fibroblast growth factor and its receptor, leading to an augmentation of pulmonary endothelial cellularity. Lastly, autoimmunity plays a role in PHTN by increased antibodies such as TRAB. These antibodies disrupt the regulatory T lymphocytes and endothelial mast cell activation. A pro-inflammatory state follows, resulting in endothelial damage and PHTN.^[1]^ The pathophysiology is shown in a flowchart (Fig. 4).

In a recent study published in 2021, Song et al found PHTN in 35% (111) patients with HTH in their cohort. In multi-logistic regression, the authors did not find any significant association of TRAB with PHTN development. However, they did report higher T4 levels to be significantly associated with the development of PHTN.^[14]^ In our review’s patient population (PHTN with HTH), TSH, T3, or T4 levels did not show any statistically significant association with outcomes of PHTN. This could indicate that although a more severe HTH is associated with PHTN, once it develops, the severity of HTH does not affect the reversibility of PHTN. Song et al also reported that PHTN might be present in HTH of any etiology, which correlates to our findings, as we found PHTN in patients with Graves, TMNG, thyroiditis, drug-induced HTH, and toxic adenoma. 95% of patients had Graves disease in their study, compared to 66.6 % in ours. Although Graves disease seems to be the most common cause of PHTN in patients with HTH, it should be kept in mind that Graves generally is the most prevalent cause of HTH overall, causing up to 80% of HTH.^[34]^

The association of PHTN in thyroid disorders may not be widely known, especially among the emergency and the internal medicine physicians who are the primary contact of patients admitted with thyroid disorders. Diagnosing PHTN due to any cause had remained a challenge, and even with increased knowledge and awareness, up to 20% of patients with symptoms of PHTN remain undiagnosed for at least 2 years before PHTN diagnosis is established.^[35]^ The prevalence of PHTN in HTH is debatable, with different values reported in the literature. This can be due to the small sample sizes of individual studies compared to the global burden of HTH or can be due to the demographically distinct course of HTH. In their study, Zhang et al found PHTN in 71% of hyperthyroid patients.^[18]^ The authors focused on the association of various thyroid antibodies with the development of PHTN and found higher levels of TRAB to be significantly associated with PHTN. They did not find any statistically significant association of antiTG AB with PHTN development.^[18]^ The association of thyroid antibodies with PHTN seems to differ in multiple studies. Marvisi et al, in their study on 34 patients, did not find any statistically significant association of thyroid antibodies with PHTN.^[9]^ On the other hand, Suguira et al, in their study on 59 patients, found TRAB to be significantly associated with PHTN (*P*-value <0.001).^[15]^ Whether the difference in results is due to the sample size variation or geographic variation in disease presentation can be answered by studying the effects of thyroid antibodies on the development of PHTN in larger cohorts. Additionally, it is vital to study the effect of these antibodies on the progression and management of PHTN. We found that patients who had positive TRAB had a slightly lower but nonsignificant response rate in PHTN to antithyroid treatment than those with negative TRAB (87.5% vs 88.8%, *P*-value: 0.6). This might indicate that TRAB is associated with the development as well as poor outcomes of PHTN. However, as the clinical difference is minimal, it might eliminate or widen if larger prospective studies are conducted. As it is proposed that autoimmunity may play a role in the pathophysiology of development of PHTN, it might have a role in its progression also and this needs validation in larger studies.

Singarayar et al published a review of 25 cases on patients with PHTN and HTH. They reported a predominantly female population (71%). Another review published by Vallabhajosula et al reported a female preponderance of 67%.^[33]^ The trend is consistent in our review of 589 patients, where females constituted 63.4% (N = 374) of the population. This indicates that despite different sample sizes, females with HTH are around 3 times more prone to develop PHTN compared to their male counterparts. This can merely be due to the fact that HTH itself is 5 times more common in females.^[36]^ Interestingly, our outcome analysis of case reports and series showed that males showed better outcomes with a 100% recovery rate in PHTN compared to an 82.6% response rate in females despite a female predominance. However, the difference was not statistically significant (*P*-value 0.17). This might be due to the small sample size in the outcome analysis (N = 34). More extensive studies can help understand gender correlations with the recovery of PHTN.

There is limited literature regarding patients with PHTN and HTH treated with medications specific for PHTN. In their study, Satoh et al retrospectively reviewed 59 patients with PHTN, out of whom 12 had thyrotoxicosis.^[13]^ These patients were treated with epoprostenol and endothelin receptor antagonists for PHTN. The authors reported a significant association between HTH development with epoprostenol treatment (OR 8.2, *P*-value = 0.08). This has not been reproduced in other studies as most of the studies conducted on PHTN and HTH did not report using specific treatment modalities approved for PHTN; almost all of the patients were managed by antithyroid therapy with reasonable response rates.^[10,20,22,24,37]^ Factors associated with a complete or partial resolution or progression of PHTN in the setting of HTH remain largely unexplored. Marvisi et al, in their second study on PHTN and HTH, reported a statistically significant association of methimazole therapy with the reduction in PAP posttreatment.^[12]^

Outcomes of PHTN in patients with HTH are generally good. Progression of PHTN is rarely encountered if HTH is managed, regardless of the modality of treatment (medication, radio ablation, or surgery). Armigliato et al reported a resolution rate of 92.3% in their cohort of 13 patients with PHTN and HTH, treated with methimazole (N = 9), raioablation (N = 1), and thyroidectomy (N = 2). Another study on 14 patients with PHTN and HTH reported a 100% resolution rate in PHTN, evident by follow-up echocardiogram showing normalization in PAP.^[21]^ In our review, we found thionamides as the most common treatment (N = 262, 44.4%), followed by surgery (N = 54, 9.1%) and radio ablation (N = 34, 5.7%). We assessed the differences in the response rates in case reports and series. It was not possible to assess the response rates to specific treatments in the larger studies added in our review, as the data could not be individualized for each patient. Overall, the response rates were good, with improved PHTN in at least >90% with each treatment modality. We did not find any statistically significant differences in the response rates of the treatment modalities. However, this is an area that needs further exploration in larger prospective studies.

Our review has some limitations, which are inherent to systematic reviews. First, we could not analyze the larger studies’ data as the data was not individualizable and we could not obtain individual data from the authors of these studies. In most cases, it was not possible to identify and relate the outcomes to individual patients in the cohorts due to the cumulative presentation of data in those studies. Therefore, statistical analysis was done only on data from case reports and series where outcomes could be analyzed based on individual patient variables. Second, a considerable number of studies did not report outcomes of HTH and PHTN. Thirdly, many patients may have had asymptomatic PHTN and this may have resulted in under reporting, making the true prevalence higher. Additionally, as this is a systematic review of previously published data, there may have been variations in the investigations performed to rule out other causes of PHTN. This can be better answered in a prospective study with a uniform methodology to rule out all other possible causes of PTHN in HTH patients. Moreover, as this is a review on previously published data, there might be unpublished work on PHTN secondary to HTH which might have affected the results stated above. An insufficient amount of systematically described prospective data impedes adequate information on the true incidence or timeline of the development of PHTN in patients with HTH. Lastly, a majority of patients did not have right heart catheterization findings reported and it was unclear whether the diagnosis was made on echocardiographic findings only or confirmed with catheterization.

This review opens doors for further prospective studies which should focus on the clinical presentation of PHTN in patients with HTH, and the various variables that have been described above to possibly have a significant role in the resolution of PHTN in the setting of HTH.

## 5. Conclusions

PHTN is not an uncommon occurrence in HTH and may be more prevalent than currently thought of. It can be a presenting feature of HTH, which is associated with better outcomes of PHTN. There is a clear female preponderance in the development of PHTN. However, resolution rates seems to be better in males. Hypertensive patients tend to have comparatively poor outcomes of PHTN in this group of patients. Although positive TRAB is associated with the development of PHTN, it does not seem to affect its outcome. Lastly, PHTN in patients with HTH does not need any specific management, and there is more than a 90% resolution rate with antithyroid therapy. Whether any specific antithyroid therapy has a better outcome in PHTN needs to be explored in larger prospective patient cohorts.

**Table 1:** Clinical features of reported hyperthyroid patients with PHTN.

Characteristics	Patients (N = 589)
Age (years) (range)	0.01–75
Gender	
Males (N with %)	173 (29.3 %)
Females (N with %)	374 (63.4 %)
Comorbidities (N with %)	50 (8.4%)
HTN	14 (2.3 %)
DM	5 (0.8%)
CHF	4 (0.6%)
CAD	1 (0.1%)
CKD	1 (0.1%)
COPD	
Features of HTH (N with %)	
A fib	20 (3.3%)
Palpitations	18 (3%)
Goiter	15 (2.5%)
Tremors	14 (2.3%)
Weight loss	12 (2%)
Ophthalmopathy	12 (2%)
Diarrhea	9 (1.5%)
Sweating	6 (1%)
Heat intolerance	3 (0.5%)
Hth investigations	
TSH (mU/L) (range)	0.0–0.09
T3 (ng/dl) (range)	0.5–771
T4 (ng/dl) (range)	1.28–20,800
TRAB + (N with %)	283 (48%)
Anti TPO + (N with %)	272(46.1%)
ANA + (N with %)	10 (1.69%)
Thyroid US + (N with %)	20 (3.3%)
RIU + (N with %)	168 (28.5%)
Etiology of HTH (N with %)	
Graves	393 (66.7%)
TMNG	99 (16.8%)
Thyroiditis	5 (0.8%)
Drug-induced	2 (0.3%)
Toxic adenoma	1 (0.1%)
nonspecific	89 (15.1%)
Treatment of Hth (N with %)	262 (44.4%)
Thionamides surgery	54 (9.1%)
Radio ablation	34 (5.7%)
Responded to 2nd line	5 (0.8%)
Outcomes of Hth (N with %)	
Euthyroid post Tx	178 (30.2%)
Persistent Hth	20 (3.39%)
Not reported	391 (66.3%)
Features of PHTN (N with %)	
Presence at initial diagnosis of HTH	83 (14%)
Dyspnea	108 (18.3%)
Peripheral edema	15 (2.5%)
Anorexia	3 (0.5%)
Cough	3 (0.5%)
Abdominal pain	2 (0.3%)
Chest pain	1 (0.1%)
Syncope	1 (0.1%)
Diagnosis of PHTN (N with %)	
Echocardiogram	589 (100%)
Cardiac Catheterization	3 (0.52%)
Treatment of PHTN (N with %)	
No specific Tx	574 (97.4%)
Diuretics	12 (2%)
CCB	1 (0.1%)
NO	2 (0.3%)
Outcomes of PHTN	
PAP before Tx (mm Hg) (range)	22.5-75
PAP after Tx (mm Hg) (range)	24-50
RVSP before Tx (mm Hg) (range)	60-80
RVSP after Tx (mm Hg) (range)	35-41
Resolution of PHTN (N with %)	98 (16.6%)
Improved PHTN (N with %)	72 (12.2%)
Persistent PHTN (N with %)	9 (1.5%)
Progression of PHTN (N with %)	0
Death (N with %)	2 (0.3%)
Not reported	408 (69.2%)

Data were not normal (assessed by Shapiro–Wilk test), except PAP before therapy. Age, TSH, T3, T4, PAP, and RVSP were reported as means or medians in larger studies, which made it difficult to cumulate them in a single value; hence, we have reported their ranges instead and density histograms.

CAD: Coronary artery disease, CHF: Congestive heart failure, CKD: Chronic kidney disease, CLD: chronic liver disease, COPD: Chronic obstructive pulmonary disease, DM: Diabetes mellitus, HTH: Hyperthyroidism, HTN: Hypertension, PAP: Pulmonary artery pressure, PHTN: Pulmonary hypertension, RIU: Radioactive iodine uptake, RVSP: Right ventricular systolic pressure, SOB: Shortness of breath, TPO: Thyroid peroxidase, TRAB: Thyroid receptor antibodies, TSH: Thyroid stimulating hormone, Tx: Treatment.

**Table 2:** Data readily available from the 3 included prospective studies on patients with PHTN with HTH.

Author, year	N	Mean Age (y)	Gender (M: F)	Features at presentation	Mean TSH (mU/L)	Mean T3 (ng/dL)	Mean T4 (ng/dL)	Tx	Euthyroid postTx	PAP before Tx	PAP after Tx	Outcomes of PHTN
Armigliato (2006)	23	45.8	8: 15	Ophthalmopathy: 7	0.009	0.86	4.6	Thionamides: 12	15	36.7	28.6	Resolved: 15
				SOB: 1				RIA: 1				Persistent PHTN: 1
								Surgery: 2				
Merce (2005)	39	52	11: 28	A. Fib: 7	0.0065	NA	4.46	NA	33	38	29	Improved PHTN: 33
												Death: 1
Siu (2007)	35	43	12: 23	NA	NA	NA	6.4	Thionamides: 30	NA	48.5	34	Improved PHTN: 1
								RIA: 5				

A. Fib: Atrial fibrillation, F: Females, M: Males, N: Number, NA: Not available, PHTN: Pulmonary hypertension, RIA: Radioactive iodine ablation, SOB: Shortness of breath, TSH: Thyroid stimulating hormone, Tx: Treatment.

### Author contributions

FA: Conceptualization, methodology, data collection, analysis and interpretation, literature review, manuscript preparation, critical review, and revisions in the manuscript

AAK: Literature review, data collection and interpretation, manuscript preparation, critical review, and revisions in the manuscript

HC and ZY: Literature review, data collection, analysis, and interpretation, manuscript preparation, critical review, and revisions in the manuscript

AU, BA, FK: Literature review and data collection

MZ, DA, and EN: Supervision, critical review, and revisions in the manuscript

All authors read and approved the final manuscript

### Acknowledgment

The Qatar National Library funded the publication of this article.
